# Metagenomic next-generation sequencing of nasopharyngeal microbiota in COVID-19 patients with different disease severities

**DOI:** 10.1128/spectrum.04166-23

**Published:** 2024-04-01

**Authors:** Waleed Aljabr, Iman Dandachi, Basma Abbas, Alaa Karkashan, Ahod Al-Amari, Dayel AlShahrani

**Affiliations:** 1 Research Center, King Fahad Medical City, Riyadh, Saudi Arabia; 2 Institute of Infection, Veterinary and Ecological Sciences, University of Liverpool, Liverpool, United Kingdom; 3 Department of Biological Sciences, College of Science, University of Jeddah, Jeddah, Saudi Arabia; 4 Department of Basic Medical Sciences, College of Medicine, Dar Al-Uloom University, Riyadh, Saudi Arabia; 5 Pediatric infectious diseases, King Fahad Medical City, Riyadh, Saudi Arabia; Children's National Hospital, George Washington University, Washington, DC, USA

**Keywords:** metatranscriptomics, COVID-19, disease severity, microbiome

## Abstract

Throughout the COVID-19 pandemic, extensive research has been conducted on SARS-COV-2 to elucidate its genome, prognosis, and possible treatments. However, few looked at the microbial markers that could be explored in infected patients and that could predict possible disease severity. The aim of this study is to compare the nasopharyngeal microbiota of healthy subjects, moderate, under medication, and recovered SARS-COV-2 patients. In 2020, 38 nasopharyngeal swabs were collected from 6 healthy subjects, 14 moderates, 10 under medication and 8 recovered SARS-COV-2 patients at the Prince Mohammed Bin Abdulaziz Hospital Riyadh. Metatranscriptomic sequencing was performed using Minion Oxford nanopore sequencing. No significant difference in alpha as well as beta diversity was observed among all four categories. Nevertheless, we have found that *Streptococcus* spp including *Streptococcus pneumoniae* and *Streptococcus thermophilus* were among the top 15 most abundant species detected in COVID-19 patients but not in healthy subjects. The genus *Staphylococcus* was found to be associated with COVID-19 patients compared to healthy subjects. Furthermore, the abundance of *Leptotrichia* was significantly higher in healthy subjects compared to recovered patients. *Corynebacterium* on the other hand, was associated with under-medication patients. Taken together, our study revealed no differences in the overall microbial composition between healthy subjects and COVID-19 patients. Significant differences were seen only at specific taxonomic level. Future studies should explore the nasopharyngeal microbiota between controls and COVID-19 patients while controlling for confounders including age, gender, and comorbidities; since these latter could affect the results and accordingly the interpretation.

**IMPORTANCE:**

In this work, no significant difference in the microbial diversity was seen between healthy subjects and COVID-19 patients. Changes in specific taxa including *Leptotrichia*, *Staphylococcus,* and *Corynebacterium* were only observed. *Leptotrichia* was significantly higher in healthy subjects, whereas *Staphylococcus* and *Corynebacterium* were mostly associated with COVID-19, and specifically with under-medication SARS-COV-2 patients, respectively. Although the COVID-19 pandemic has ended, the SARS-COV-2 virus is continuously evolving and the emergence of new variants causing more severe disease should be always kept in mind. Microbial markers in SARS-COV-2 infected patients can be useful in the early suspicion of the disease, predicting clinical outcomes, framing hospital and intensive care unit admission as well as, risk stratification. Data on which microbial marker to tackle is still controversial and more work is needed, hence the importance of this study.

## INTRODUCTION

SARS-COV-2 is a virus that caused major worldwide health issues since its emergence in 2019. SARS-COV-2 belong to the beta genus of the coronaviridae family and is characterized by an enveloped, positive sense single stranded RNA genome (1). Until now, this virus is continuously evolving and circulating all over the world. There are currently three circulating variants of interests: XBB.1.5, XBB.1.16, EG.5 and seven circulating variants under monitoring: BA.2.75, CH.1.1, XBB, XBB.1.9.1, XBB.1.9.2, XBB.2.3, and BA.2.86 (2). Up to date, there are up to 694,122,809 cases worldwide and 6,910,119 deaths (3). SARS-COV-2 causes a respiratory disease that ranges from an asymptomatic infection to pauci-symptomatic to critical illness (4). Clinical manifestation of SARS-COV-2 is often characterized by systemic symptoms including shortness of breath, fever, cough, fatigue, and chills. Severity and complications can present in the form of pneumonia and heart, liver or respiratory failure, respectively (5). This is in addition to the possibility of developing acute respiratory distress syndrome as well as multi-organ failure (6, 7). Severe SARS-COV-2 infection is characterized by a cytokine like syndrome due to the activation of the innate immune response (8). Studies have suggested that this cytokine storm is a major cause of disease severity as well as death in SARS-COV-2 cases (9). Indeed, it has been demonstrated that recognition/treatment of cytokine storm might be crucial for the reduction of mortality rates in severely infected COVID-19 patients (8, 10). For instance, several factors play a role in the disease severity including patient immunity status, age, gender, pre-existing comorbidities such as diabetes, hypertension, chronic kidney, or cardiovascular diseases (11, 12).

Respiratory microbiota refers to the group of microorganisms that inhabit the respiratory tract from nostrils to alveoli (13). Next generation sequencing techniques revealed that the upper respiratory tract is a dynamic ecosystem and its microbiota plays vital roles inside the human body (14). These roles include metabolic functions, shaping local immune responses, maintaining mucosal homeostasis as well as regulation of adaptive responses (15). Colonization of the upper respiratory tract starts at birth and is shaped based on three modalities: mode of delivery, antibiotics, and environment (habitat, diet, pollutants and allergens) (16). Moreover, it has been found that respiratory viruses can also alter the upper and lower respiratory microbiota, causing accordingly increased disease severity due to the resulting increased abundance of opportunistic pathogens (17, 18). For example, several studies have shown a correlation between the composition of the nasopharyngeal microbiota and patients’ susceptibility to several respiratory viruses including respiratory syncytial virus, influenza A/B and rhinoviruses (14). In their study, Vissing et al. found that colonization of the neonates’ airway with *Haemophilus influenzae*, *Moraxella catarrhalis*, and *Streptococcus pneumoniae* in neonates is associated with an increased risk of bronchiolitis or pneumoniae in the first 3 years of life (19). Another study reported that lower susceptibility to influenza H3N2 was associated with 10-fold abundance increase in *Streptococcus* spp. as well as *Prevotella salivae*. On the contrary, 10-fold abundance increase in *Prevotella* spp was associated with an increased susceptibility to Influenza B (20). As for SARS-COV-2, several studies have explored differences in the nasopharyngeal microbiota composition between SARS-COV-2 infected patients and healthy subjects. The results of these studies were controversial, with some papers finding significant differences between infected and non-infected patients, while others not finding any significant results (15). The aim of this study is thus to explore the nasopharyngeal microbiota composition in SARS-COV-2 patients with moderate disease, under medication, recovered, and healthy subjects.

## MATERIALS AND METHODS

### Samples and data collection

Thirty-eight nasopharyngeal swabs were collected from patients who were above 15 years old and were admitted at the Prince Mohammed Bin Abdulaziz Hospital Riyadh, during 2020 and are suspected for the COVID-19 disease. These included 6 healthy, 14 with moderate disease, 10 under-medications, and 8 recovered. Patients were characterized as healthy if they had no history of respiratory illnesses, smoking, or obesity, characterized as moderate if they are hospitalized in a non-ICU ward and did not start treatment (samples collected between 4 and 9 days [average of 6.5 days] from the day of reporting COVID-19 positive), as patients under medication if they are hospitalized in a non-ICU ward and have started medical treatment (samples collected between 10 and 14 days [average of 12 days] from the day of reporting COVID-19 positive), and as recovered if the sample was collected between 14 and 20 days [average of 17 days] after the first COVID-19 positive test result. Moreover, patients were characterized as youth if their age ranged from 18 to 35 years old, middle age if they are 36–55 years old and older if their age was above 55 years (21). The demographic data including age, gender and nationality were retrieved for all included subjects.

### SISPA

For the identification of viral as well as bacterial transcripts present in the NPs samples, Sequence-independent, single-primer amplification (SISPA) was conducted, as previously described (22). In brief, RNA was reverse transcribed into first strand cDNA using Sol-A primer 5′-GTTTCCCACTGGAGGATA-N9-3’, under the following conditions: 65°C, 5 min; put on an ice block for 1 min; then 23°C, 10 min; 55°C, 10 min; 80°C, 10 min. For the second strand cDNA synthesis, the conditions were as follows: 95°C, 3 min; put on an ice block for 1 min; then 37°C, 60 min. cDNA products were than purified using the AMPure XP beads (Beckman Coulter, USA), as per the manufacturer instructions. Thereafter, using Sol-B primer 5′-GTTTCCCACTGGAGGATA-3′, the second strand of cDNA was amplified as follows: 98°C for 30 s; 30 cycles of 98°C for 10 s, 54°C for 30 s, 72°C for 1 min; 72°C for 10 min. Amplified cDNA was purified using AMPure XP beads (Beckman Coulter, USA). Qubit double-stranded DNA (dsDNA) high-sensitivity (HS) assay (Q32851; Invitrogen) was then used to quantify amplified products before proceeding to sequencing.

### Minion sequencing

For the metatranscriptomic sequencing, the PCR tiling of COVID-19 virus, Version: PTC_9096_v109_revD_ 06Feb2020 was used. Generated amplicons from SISPA products from individual patients were pooled and purified in a 1:1 ratio with AMPure XP beads (A63882; Beckman Coulter) as per the manufacturer’s instructions. The library was then prepared as per the sequencing by ligation protocol with native barcodes for multiplexing (SQK-LSK109; Oxford Nanopore Technologies). Thereafter, the sequencing library was added to a flow cell connected to a MInIT device and sequencing was initiated via MinKNOW.

### Bioinformatic analysis

Using Guppy (ONT-guppy-cpu-4.4.1-win64), Fast5 files were based-called. Using Kraken2, metatranscriptomic reads were assessed (23). The abundance of genus, and phyla was estimated using Bracken (24). Alpha and beta diversity were then calculated using Krakentools (25). Using Rstudio and the calculated beta diversity, principal component analysis (PCA) and accordingly ggplot2 were performed (26). Non-metric multidimensional scaling was also performed (27). Using also Krakentools, Kraken reports were converted to a MetaPhlan-style-report and a heatmap was generated showing the most abundant 15 species using “generate heatmap” (28). Shapiro-Wilk test was used to determine the normality of our data (29). When data were normally distributed, ANOVA test was conducted to explore differential abundance of taxa and alpha diversity differences between different categories. On the other hand, Kruskal–Wallis test was used when data were not normally distributed. Furthermore, linear discriminant analysis with effect size was conducted to identify family and genera signatures in each category (30).

## RESULTS

### General characteristics of the studied population

Of the included thirty-eight subjects, the majority were males (23, 60.5%), non-Saudi (28, 73.6%) and above 55 years of age (18, 47%). Non-Saudi nationalities included Egyptian, Pakistani, Yemeni, and Palestinian (Table 1).

**TABLE 1:** Demographic characteristics of included patients^*a*^

Label	Age	Gender	Nationality	Viral load (Ct value)
9O-H	28	Male	Yemeni	NEG
26O-H	34	Male	Yemeni	NEG
27O-H	67	Male	Syrian	NEG
31O-H	44	Male	Saudi	NEG
52O-H	44	Male	Indonesian	NEG
55O-H	21	Female	Saudi	NEG
1O-M	36	Female	Palestinian	NEG
3O-M	62	Male	Afghan	NEG
4O-M	41	Male	Filipino	30
6O-M	69	Male	Canadian	NEG
10O-M	50	Male	Saudi	NEG
16O-M	56	Female	Sudanese	30.5
19O-M	60	Male	Saudi	NEG
21O-M	38	Male	Pakistani	NEG
25O-M	70	Male	Yemeni	NEG
38O-M	37	Female	Nepalese	NEG
40O-M	55	Male	Yemeni	NEG
44O-M	58	Male	Saudi	36.6
54O-M	64	Female	Saudi	NEG
56O-M	56	Female	Bangladesh	NEG
8O-UM	54	Male	Egyptian	NEG
15O-UM	43	Male	Palestinian	26
17O-UM	68	Female	Palestinian	33.6
35O-UM	67	Male	Saudi	NEG
37O-UM	39	Male	Egyptian	NEG
43O-UM	40	Female	Egyptian	NEG
46O-UM	45	Male	Pakistani	NEG
47O-UM	75	Female	Saudi	NEG
57O-UM	61	Female	Saudi	NEG
64O-UM	29	Male	Egyptian	NEG
13O-R	61	Female	Pakistani	NEG
14O-R	56	Female	Indian	NEG
23O-R	61	Male	Egyptian	NEG
28O-R	41	Female	Moroccan	NEG
34O-R	50	Male	Filipino	NEG
41O-R	60	Female	Saudi	NEG
58O-R	41	Male	Bangladesh	NEG
59O-R	65	Female	Jordanian	NEG

^*a*^ O = oral, H = healthy, M = moderate, UM = under medication, R = recovered, NEG = negative ct value.

### Diversity of the nasopharyngeal microbiota within and between different categories

**Fig 1:**
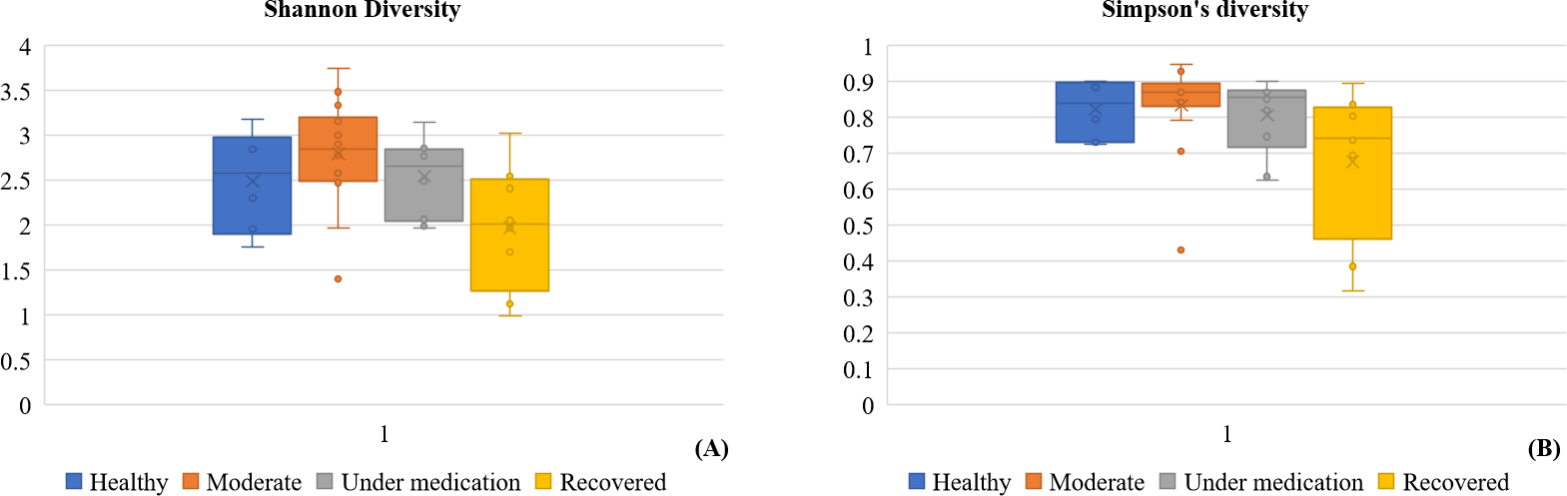
Alpha diversity of all samples, based on (**A**) Shannon’s and (**B**) Simpson’s diversity index.

**Fig 2:**
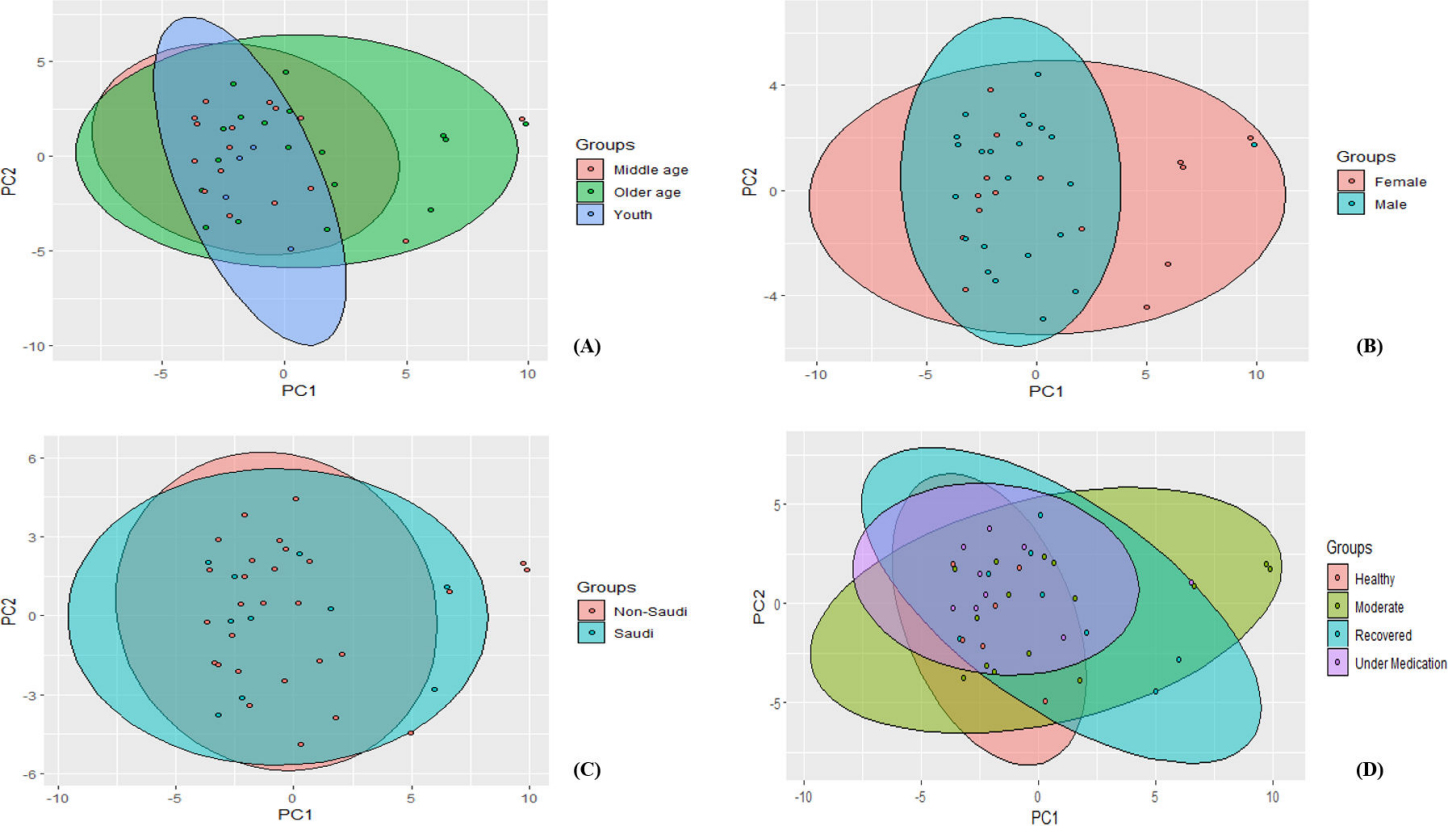
Principal component analysis of COVID-19 samples based on (**A**) age, (**B**) gender, (**C**) nationality, and (**D**) disease severity.

**Fig 3:**
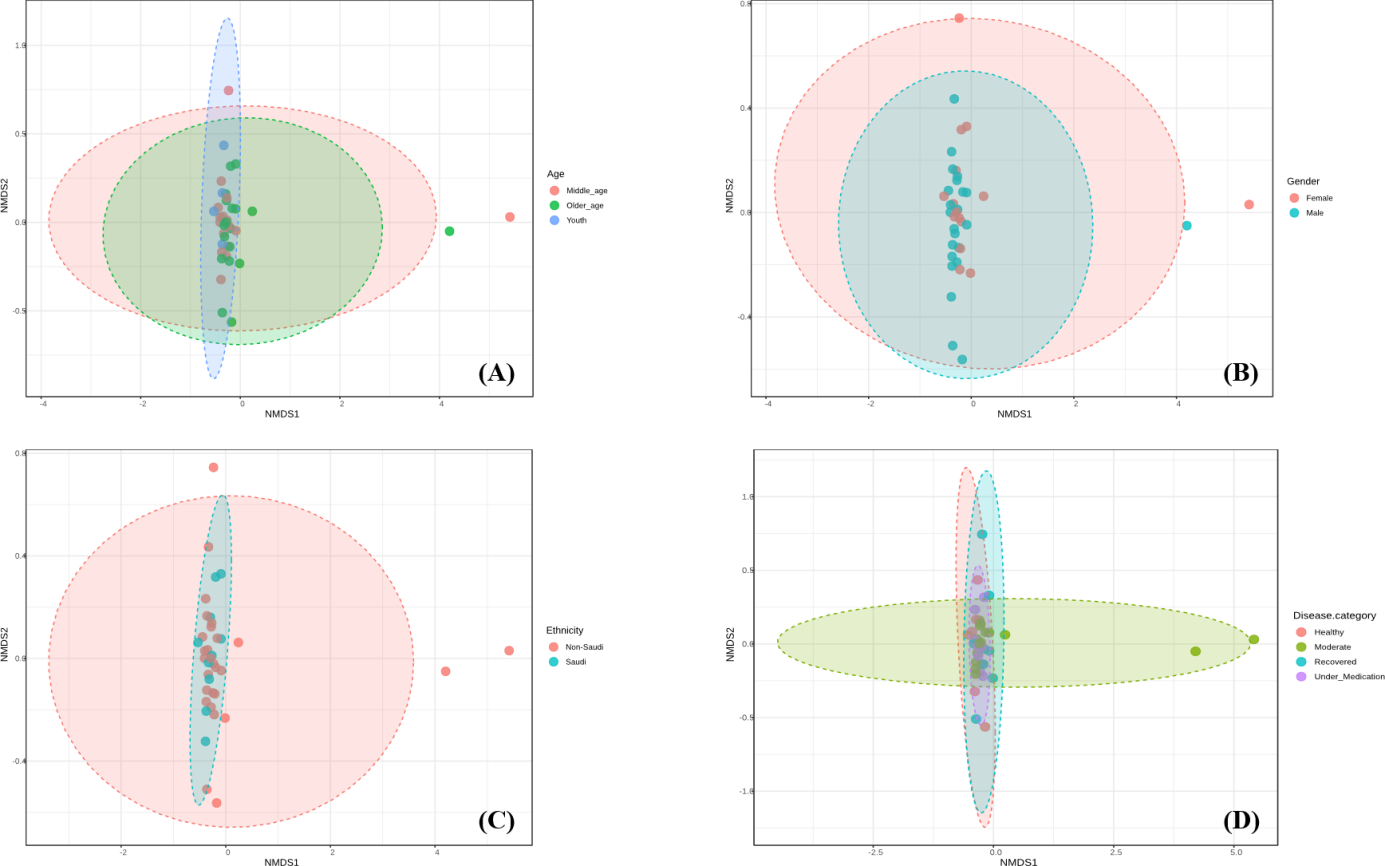
Non-metric multidimensional scaling analysis of COVID-19 samples based on (**A**) age, (**B**) gender, (**C**) nationality, and (**D**) disease severity.

A total of 4,131,662 reads were obtained from the SISPA metatranscriptomic sequencing with a mean quality score ≥Q17. An average of 108,728 reads was obtained per sample. Based on the Shannon index, mean alpha diversity was significantly higher in moderate patients compared to recovered patients (*P* < 0.05) (Fig. 1A). However, alpha diversity based on Simpson’s diversity index showed no different significant differences in between all four categories (*P* > 0.05) (Fig. 1B). On the other hand, principal component analysis and non-metric multidimensional scaling showed that all samples did not form separate clusters neither at the disease category level, nor at the age, gender or nationality levels (Fig. 2 and 3).

### Differential abundance of taxa between healthy and COVID-19 patients

**Fig 4:**
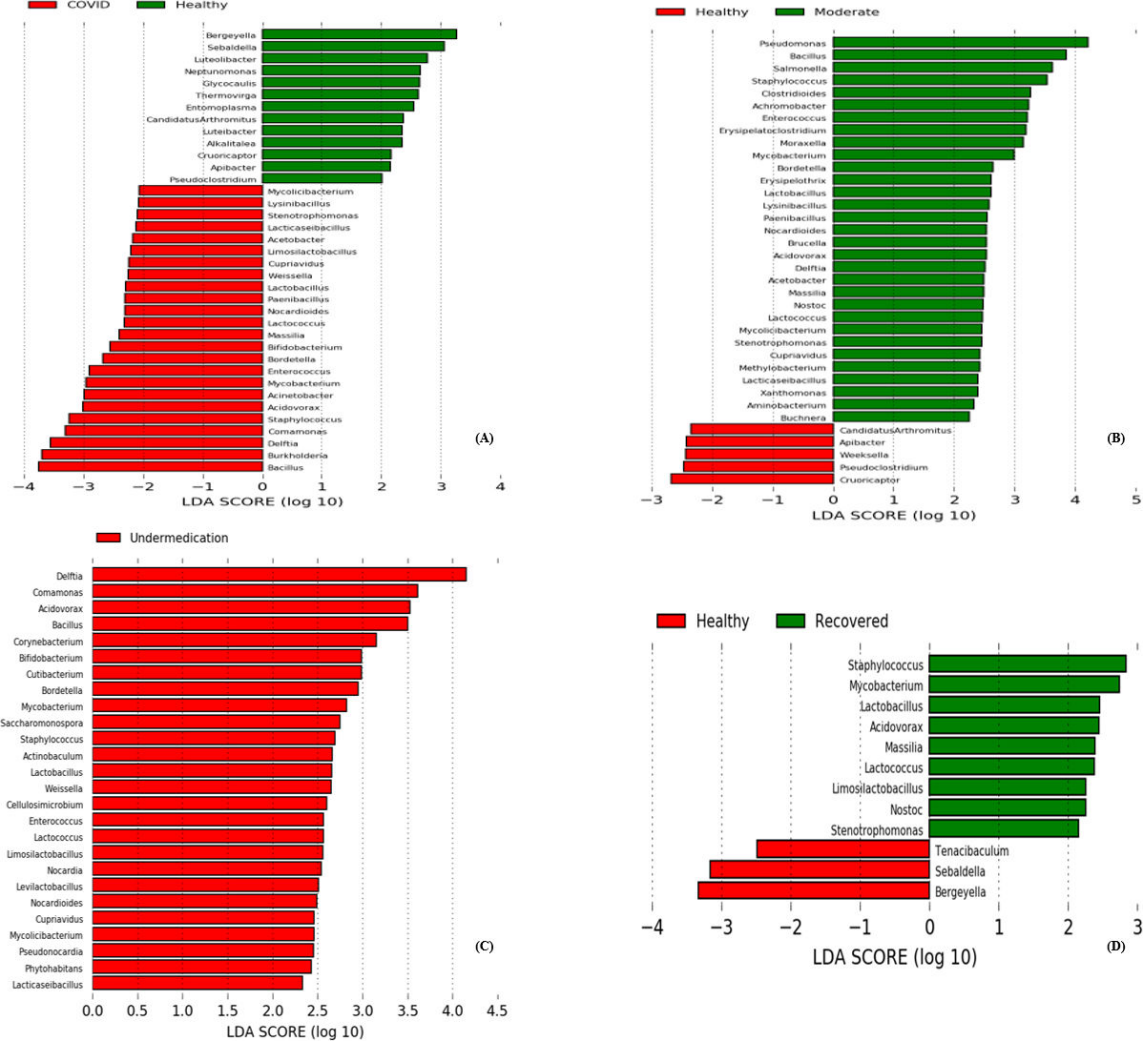
Linear discriminant analysis of effect size of nasopharyngeal microbiota at the genera level. Genera with significant (*P* < 0.05) difference in relative abundance were identified when comparing (**A**) healthy vs COVID patients which include moderate, under-medication and recovered, (**B**) healthy vs moderate patients, (**C**) healthy vs under-medication patients, (**D**) healthy vs recovered patients.

**Fig 5:**
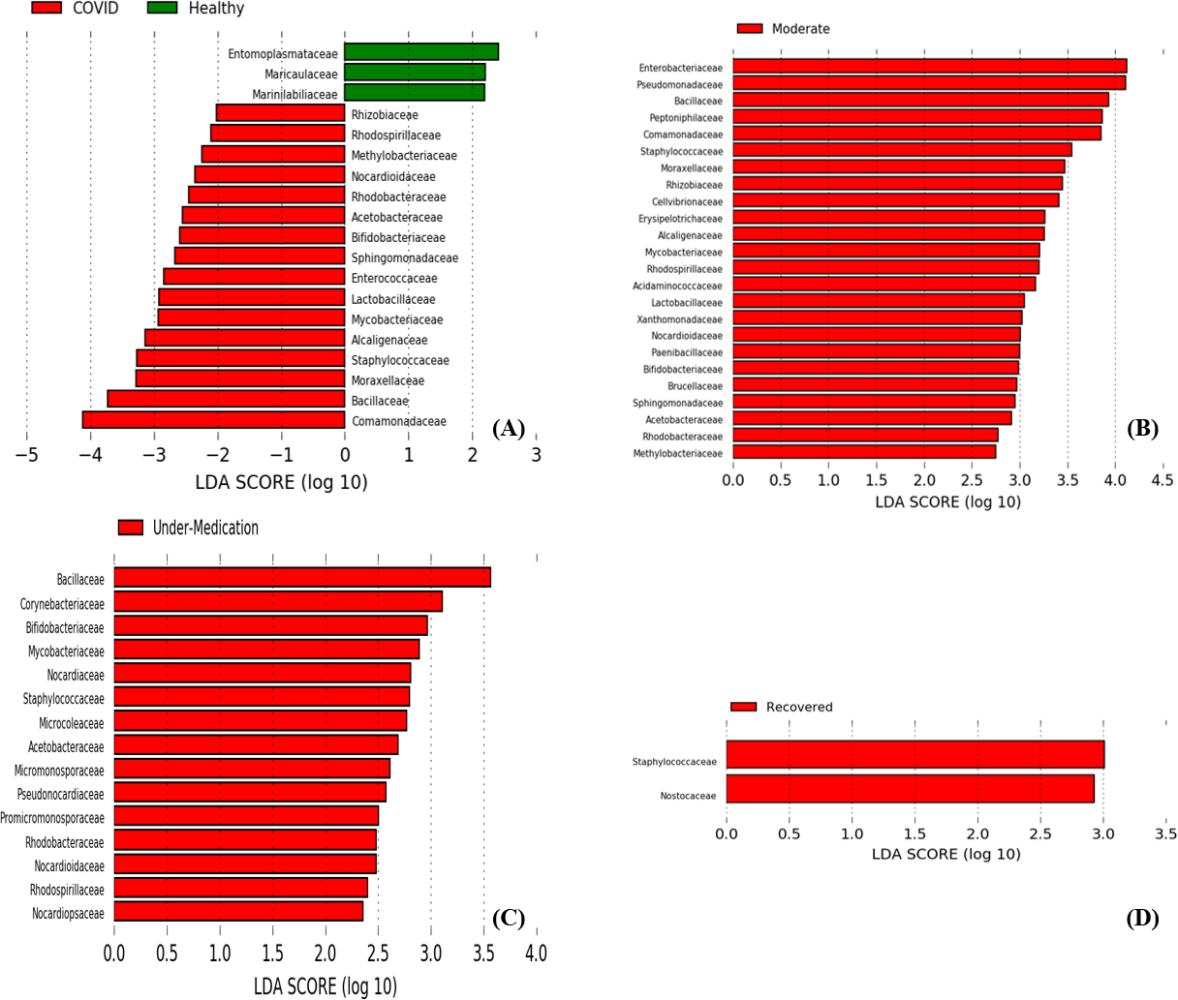
Linear discriminant analysis of effect size of nasopharyngeal microbiota at the family level. Families with significant (*P* < 0.05) difference in relative abundance were identified when comparing (**A**) healthy vs COVID patients which include moderate, under-medication and recovered, (**B**) healthy vs moderate patients, (**C**) healthy vs under-medication patients, (**D**) healthy vs recovered patients.

Linear discriminant analysis of effect size showed that when comparing healthy and moderate subjects, *Pseudomonas, Bacillus, Salmonella,* and *Staphylococcus* were the genera that distinguish the moderate group (Fig. 4B). *Delftia, Comamonas, Acidovorax, Bacillus* and *Corynebacterium* were the signature genera in patients’ under-medication when compared to healthy subjects (Fig. 4C). On the other hand, when recovered patients were compared to healthy subjects, *Staphylococcus, Mycobacterium* were the most common in the recovered category (Fig. 4D). When all healthy subjects were compared to COVID patients, it was found that *Bacillus, Burkholderia, Delftia, Comamonas,* and *Staphylococcus* were the microbial signatures in COVID-19 patients (Fig. 4A). At the family level, *Comamonadaceae, Bacillaceae, Moraxellaceae,* and *Staphylococcaceae* were the most commonly observed in COVID-19 patients when compared to healthy subjects. On the other hand, when healthy subjects were compared to moderate, under-medication and recovered patients each apart, it was found that *Enterobacteriaceae, Pseudomonadaceae, Bacillaceae, Peptoniphilaceae,* and *Comamonadaceae* were the most common in moderates, *Bacillaceae, Corynebacteriaceae, Bifidobacteriaceae,* and *Mycobacteriaceae* in those under-medication and *Staphylacoccaceae* and *Nostacaceae* in recovered (Fig. 5).

### Differences in nasopharyngeal microbiota composition at the phylum and genus levels

**Fig 6:**
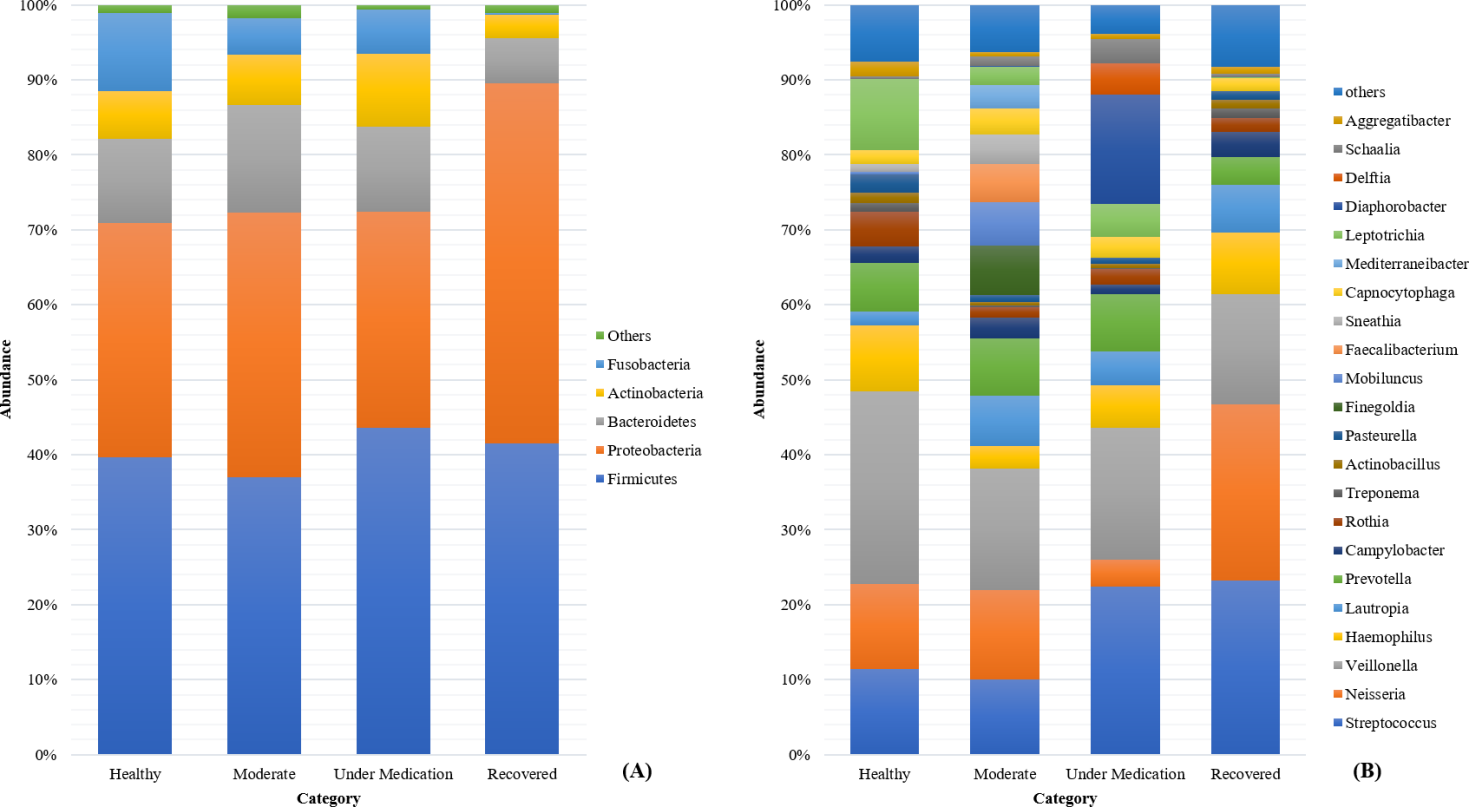
Percentage of relative abundance of the most common (**A**) phylum detected in all categories, (**B**) genera detected in all categories.

As shown in Fig. 6A, *Firmicutes, Proteobacteria*, *Bacteroidetes,* and *Actinobacteria* dominated across all categories, with no statistical difference in the abundance of these phylum being observed (*P* > 0.05). At the genera level, *Streptococcus, Neisseria, Veillonella,* and *Haemophilus* dominated in healthy, moderate, under-medication and recovered patients (Fig. 6B). Similar to the phylum, no statistical difference was observed in the abundance of the genera between all four categories (*P* > 0.05). This is except for *Leptotrichia* where its abundance was significantly higher in healthy subjects compared to recovered patients (*P* < 0.05).

### Top abundant bacterial species detected in studied subjects

**Fig 7:**
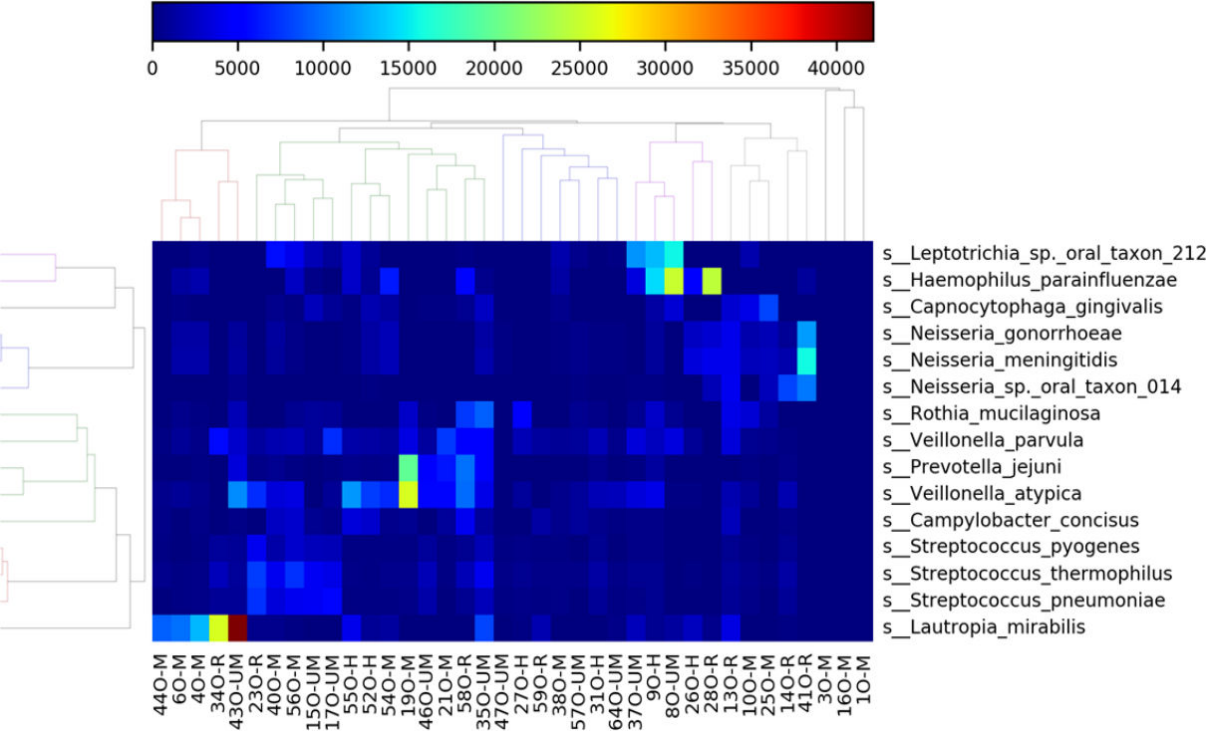
Heatmap showing the most common species in all samples included in the study. The color bar represents the abundance of species in each sample.

**Fig 8:**
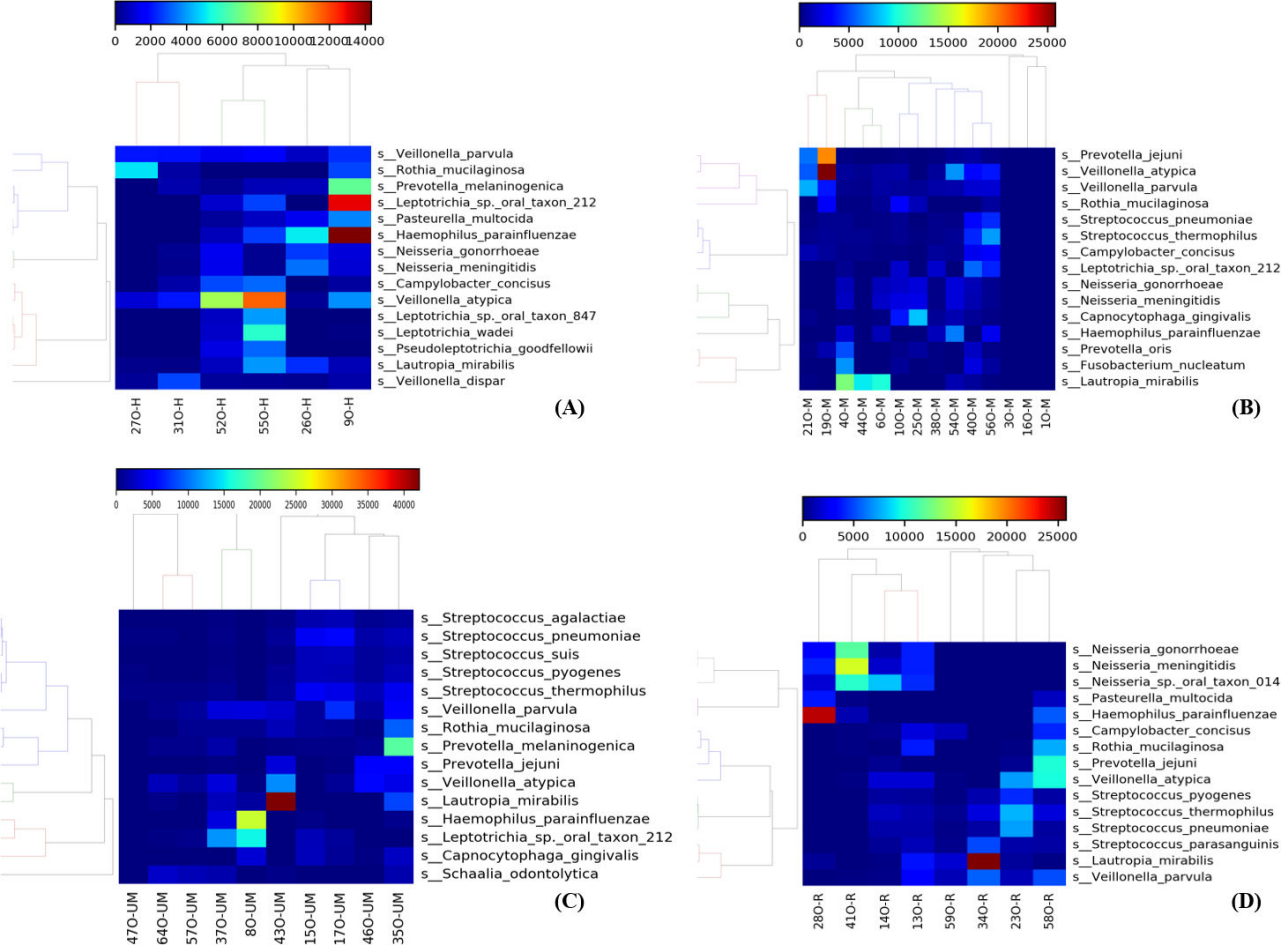
Heatmap showing the most common species across in (**A**) healthy subjects, (**B**) moderate subjects, (**C**) under-medication subjects, (**D**) recovered subjects. The color bar represents the abundance of species in each sample.

Metatranscriptomic analysis revealed that the most common species detected in all nasopharyngeal samples were *Leptotrichia sp, Haemophilus parainfluenzae*, *Capnocytophaga gingivalis, Neisseria species, Rothia mucilaginosa, Veilonella parvula, and Prevotella jejuni* (Fig. 7). More specifically, in healthy subjects, *Prevotella melaninogenica, Pasteurella multocida*, and *Neisseria species* were also among the most commonly detected. In moderate patients, *Veilionella atypica, S. pneumoniae, Streptococcus thermophilus,* and *Campylobacter concius* were among the most common. On the other hand, species most commonly observed in patients’ under-medication were *Streptococcus species* including *agalactiae, pneumoniae, suis, pyogenes, thermophilus,* in addition to *P. melaninogenica.* In recovered patients, *Neisseria species* were the most commonly observed including *gonorrhoeae*, *meningitidis*, as well as others such as *P. multocida, C. concius,* and *Veilionella atypica* (Fig. 8).

### Fungal and viral microbiome detected in this study

**Fig 9:**
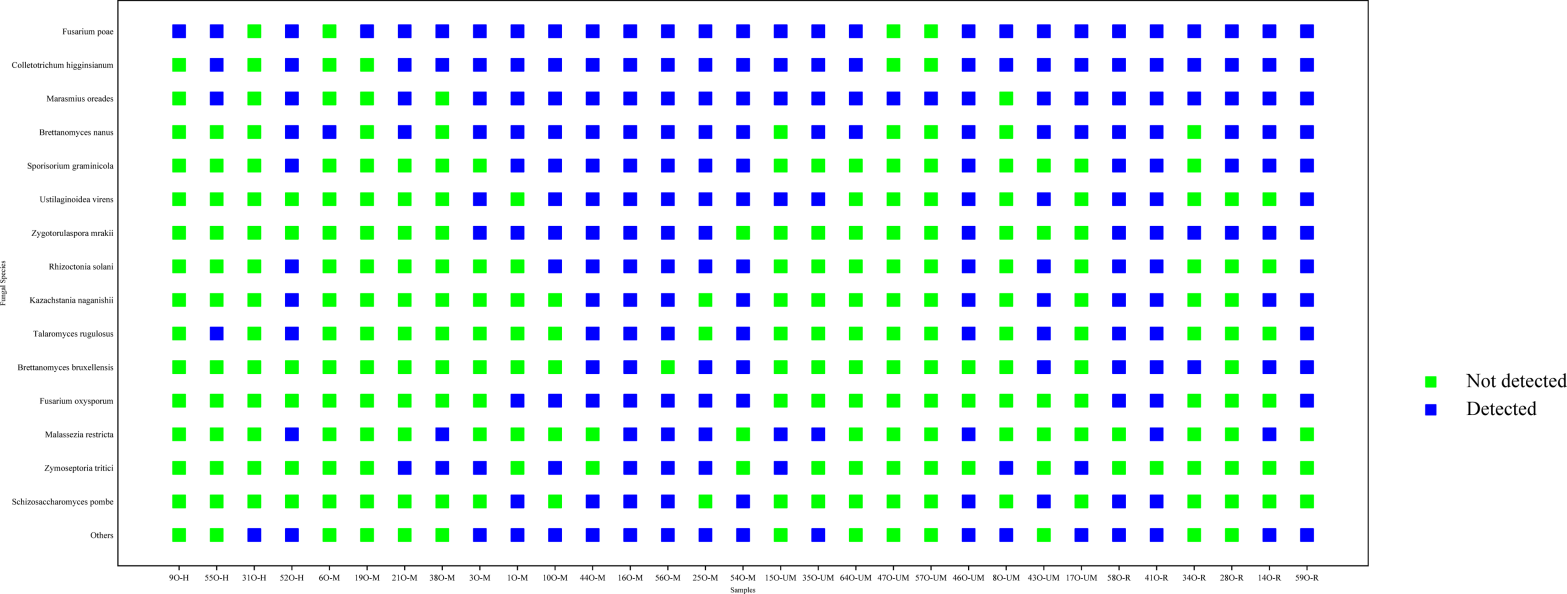
Most common fungal species detected in all samples.

Fungi were detected in thirty-one out of thirty-eight samples in this study. The most common detected fungal species were *Fusarium poae*, *Colletotrichum higginsianum*, *Marasmius oreades*, *Brettanomyces nanus* and *Sporisorium graminicola* (Fig. 9). On the other hand, the only virus detected was the SARS-COV-2, which was detected in one moderate patient and one under medication.

## DISCUSSION

Since SARS-COV-2 is a respiratory infection, dysbiosis in the microbiota of the upper airways may play a role in the disease severity in infected patients (31). Whether infection with SARS-COV-2 is responsible for changes in the nasopharyngeal microbiota compared to healthy patients or whether disease severity is associated with dysbiosis, is still controversial in the literature. In our study, no significant difference in alpha diversity between healthy and COVID-19 subjects was observed. The only finding was that moderate patients had higher mean of alpha richness compared to recovered patients; with this latter being only significant based on the Shannon index. Similarly, principal component analysis and NDMS showed no separate clusters between the four categories. Our results are in accordance with a recent study conducted in Saudi Arabia where the difference in alpha as well as beta diversity between COVID-19 positive and control subjects was not significant (32). Indeed, although, there are some studies that reported similar findings when comparing positive and negative SARS-COV-2 patients (33, 34); the majority of reports have found significant differences in alpha as well as beta diversity. For instance, in their study, Galperine et al. found that compared to healthy controls, alpha diversity decreased over time in the COVID-19 group (35). Another study reported similar findings where microbial diversity was lower in SARS-COV-2 patients and that differences in the microbial communities were linked to disease severity (36). We speculate, that one reason for results differences between studies could arise from different experimental designs, sample size, population characteristics as well as the definition used to characterize different levels of the disease severity, as well as the variant type. In Saudi Arabia, alpha and beta variants were found in genomes obtained between December 2019 and August 2021 (37). The delta variant, on the other hand, was prominent in a study conducted by Alahmad et al (38).

In terms of phyla composition, we have found no difference between healthy subjects and COVID-19 patients. As reported by several studies in the literature, regardless of the category group, the nasopharyngeal microbiota of studies subjects was found to be dominated by *Firmicutes*, *Proteobacteria*, *Bacteroidetes,* and *Actinobacteria* (39, 40). At the genus level, only the *Leptotrichia* was significantly higher in healthy compared to recovered SARS-COV-2 patients. In their study, Nardelli et al. found that in the nasopharyngeal microbiota of un-infected patients, a significant increase in the relative abundance of the genera *Leptotrichia, Fusobacterium,* and *Haemophilus* was noted compared to those un-infected (41). Similarly, another study has found that the abundance of the *Leptotrichiaceae* family was significantly higher in the nasopharyngeal microbiome of SARS-COV-2 patients compared to those un-infected (42). On the contrary, Gao et al. found that the genera *Leptotrichia* was significantly increased in the oropharyngeal microbiome of COVID-19 patients (43).

Interestingly, in our study, when comparing healthy subjects to COVID-19 patients, *Staphyloccocus* was a common microbial marker in all three categories. Also, heatmaps showed the presence of *Streptococcus spp* being among the top 15 abundant species detected in moderate, under-medication and recovered patients but not in healthy subjects. For instance, a study conducted in Italy, found a correlation between the high abundance of “super-pathogenic” bacterial species including *Staphylococcus aureus*, *S. pneumoniae*, *Acinetobacter baumannii*, *Pseudomonas aeruginosa,* and *Klebsiella pneumoniae* in SARS-COV-2 infected patients versus negative control group (44). Co-infections, superinfections and their association with poor outcomes and accordingly increased mortality and/or increased length of hospital stay as well as higher frequency of ICU admission, in SARS-COV-2 patients has been frequently reported in the literature. These include infections with *S. aureus, S. pneumoniae* and *K. pneumoniae* (45). Indeed, these species can cause respiratory illness by their own; accordingly, it can be stated that dysbiosis of the nasopharyngeal microbiota due to SARS-COV-2 and the subsequent increase in the relative abundance of pathogenic bacteria could be responsible for the disease severity in infected patients. Moreover, in our study, *Corynebacterium* was found to be associated with patients’ under-medication only. Some studies associated the increased abundance of this genera with more severe COVID-19 disease (46, 47); while others found that its abundance decreases as the severity of the illness increases (44, 48). For example, in their study, Mostafa et al. found that SARS-COV-2 positive patients have decreased abundance of *Corynebacterium accolens* compared to negative patients (49). On the other hand, Liu et al. reported the development of *C. accolens* associated ventilator associated pneumonia in a patient with respiratory failure due to COVID-19 (50). Interestingly, it has been suggested that this *Corynebacterium species* antagonize the colonization of the nasal cavity with *S. pneumoniae* (51, 52). Another interesting finding in our study, is the detection of *C. gingivalis* among the top most abundant fifteen species. Although considered part of the normal oral as well as nasopharyngeal microbiota of humans, species of the *Capnocytophaga* genus are not prominent members and are infrequently isolated from clinical samples (53). For instance, species such as *C. gingivalis, Capnocytophaga sputigena, Capnocytophaga granulosa,* and others are considered periodontal pathogens, and can also cause other infections including septicemia, endocarditis, osteomyelitis and soft tissue infections (53–56).

It is worth mentioning that in this study the viral yield was low, with SARS-COV-2 being detected only in two patients. For instance, the SISPA protocol used in this study has been previously proven to be effective in identifying various viruses including Chikungunya, Dengue and Lassa viruses (57, 58). We speculate that one reason for our finding is the low ct values and viral loads in the collected nasopharyngeal samples. This is combined with the choice of NPs that might not have allowed the detection of the common respiratory as well as relatively the fungal microbiota. Nevertheless, there are currently several molecular assays that assist in the detection of these microorganisms (59–61). Another possible reason is the large bacterial sequencing background that did not allow for the sequencing of viruses to a sufficient coverage and read depth. Indeed, in their study, Abdulrahman et al. found that upon enrichment of the SISPA-metagenomic oxford nanopore sequencing protocol with SARS-COV-2 leader sequences, coronavirus genomic as well as active microbiome information could be obtained (62).

Our study had several limitations. The first one is the low number of control samples. For instance, the uneven distribution of patients among different categories could have affected our results and so our conclusion should be carefully considered. The second one is the incomplete metadata (demographic such as obesity, history of smoking, as well as clinical characteristics including variant type) for the studied patients which hindered the full inferral analysis with the metatranscriptomic results. In addition, the uneven distribution of gender and age between different categories could have affected our results. However, since the PCA and NDMS analysis showed no clustering neither at the gender level, nor at the age, or disease category; we speculate thus that this unevenness has possibly not affected our results.

In summary, our study revealed minor differences in the nasopharyngeal microbiota composition between healthy individuals and COVID-19 patients. These differences were mostly seen at the genus level. Risk of co-infection or super-infection was seen with the abundance of pathogenic species in COVID-19 but not in healthy patients. More studies are needed to explore the differential composition of the nasopharyngeal microbiota between healthy and infected subjects while stratifying for age, gender and comorbidities with equal category size. This is because these factors could have played a role in the controversial observation of some results.

## AFTER EPUB

*This article was published on 1 April 2024 with errors in the abstract and Fig. 9. The errors were corrected in the current version, posted on 2 May 2024.*
